# Reduced Ejection Fraction of the Systemic Right Ventricle and Severe Tricuspid Regurgitation: Medication or Surgery?

**DOI:** 10.3390/jcdd12120482

**Published:** 2025-12-08

**Authors:** Anton V. Minaev, Timur Y. Danilov, Diana P. Paraskevova, Vera I. Dontsova, Inna I. Trunina, Viktor B. Samsonov, Sofya M. Tsoy, Alexander S. Voynov, Julia A. Sarkisyan

**Affiliations:** 1 Congenital Heart Diseases Department, Federal State Budgetary Institution “National Medical Research Center of Cardiovascular Surgery Named After A.N. Bakulev”, Ministry of Health of the Russian Federation, Rublevskoe Shosse 135, 121552 Moscow, Russia; 2 Cardiology Department, Moscow State Budgetary Healthcare Institution “Children’s City Clinical Hospital Named After Z.A. Bashlyaeva, Moscow City Health Department”, Schelyapovoy St. 11, 123098 Moscow, Russia; 3 Consultative and Diagnostic Department, State Budgetary Healthcare Institution “City Polyclinic No. 67 of the Moscow Department of Healthcare”, Varshavskoye Shosse 19, 117105 Moscow, Russia; 4 Cardiology Department, State Budgetary Healthcare Institution “City Polyclinic No. 220 of the Moscow Department of Healthcare”, Filevsky Bulvar 18, 121601 Moscow, Russia

**Keywords:** tricuspid insufficiency or regurgitation, tricuspid replacement, systemic right ventricle, reduced ejection fraction, heart failure

## Abstract

(1) Background: The systemic right ventricular (SRV) dysfunction and severe tricuspid regurgitation (TR) remain significant challenges in patients with congenitally corrected transposition of the great arteries (ccTGA) or following atrial switch procedures. Currently, there is no established, evidence-based medical therapy specifically designed for SRV failure, and treatment approaches are largely extrapolated from left ventricular heart failure (HF) guidelines. This therapeutic gap highlights the need for tailored pharmacologic strategies and optimized perioperative management in this unique population. The optimal timing of surgical intervention and the role of modern HF therapy are still under active investigation. (2) Methods: We present a case series of four patients (three adults and one child) with SRV dysfunction and severe TR, who underwent staged treatment consisting of optimized medical therapy followed by surgical tricuspid valve (TV) replacement. Medical therapy included positive inotropes, sacubitril/valsartan, sodium-glucose co-transporter 2 inhibitors (iSGLT2), beta-blockers, mineralocorticoid receptor antagonists (MRAs), and loop diuretics. (3) Results: All patients demonstrated clinical and hemodynamic improvement prior to surgery, with an increase in systemic ventricular ejection fraction (SVEF > 40%) and cardiac index. TV replacement was performed with favorable early postoperative outcomes and preserved ventricular function at mid-term follow-up. No mortality or major adverse events occurred during follow-up. One case of acute cystitis was associated with dapagliflozin. In all patients, postoperative SVEF remained >40%, and no recurrence of significant TR was observed. (4) Conclusions: A stepwise approach combining modern heart failure therapy and elective TV replacement in patients with SRV dysfunction and TR is safe and effective. Preoperative optimization leads to improved ventricular function and may enhance surgical outcomes. These findings support the integration of contemporary pharmacotherapy in the management strategy for SRV failure.

## 1. Introduction

The systemic right ventricular (SRV) dysfunction and tricuspid regurgitation (TR) represent common and serious complications in patients with congenitally corrected transposition of the great arteries (ccTGA) and transposition of the great arteries (TGA) after atrial switch surgery (Mustard or Senning type). These congenital defects are rare, with an estimated birth prevalence of ccTGA ranging from 0.02 to 0.07 per 1000 live births and TGA occurring in approximately 0.2–0.3 per 1000 live births [1]. Despite advances in congenital heart surgery, a significant proportion of these patients develop heart failure (HF) in adulthood, primarily due to chronic systemic pressure overload on the morphological right ventricle. Modern pharmacotherapy for heart failure with reduced ejection fraction (HFrEF) in the general population has been well defined, but evidence-based treatment strategies for SRV failure in ccTGA or TGA patients are still lacking. The current recommendations are largely extrapolated from left ventricular HF studies, while targeted trials remain absent. On the other hand, progressive TR leads to SRV dysfunction and requires surgery. There is a pressing need to define optimal medical management in this population, particularly in the perioperative setting. This case series highlights a staged therapeutic approach—combining optimized HF therapy and TV replacement—in patients with SRV dysfunction, demonstrating encouraging functional recovery and surgical outcomes.

## 2. Case Reports

### 2.1. Case 1

**Figure 1:**
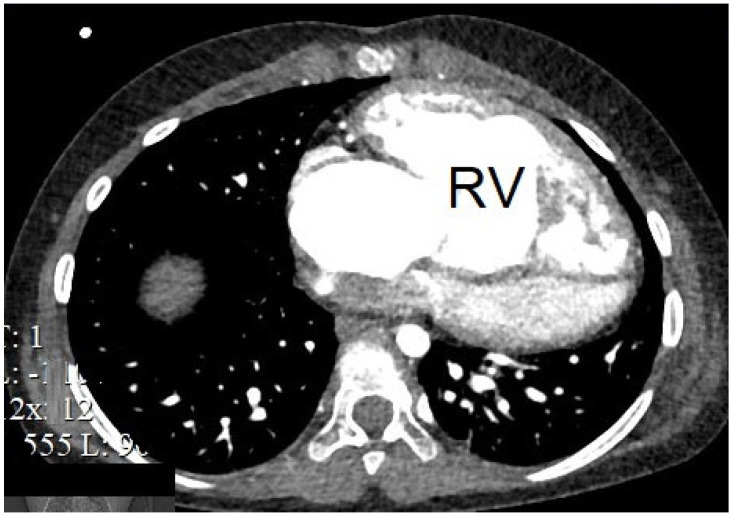
A computed tomography scan showed a dilated systemic right ventricle (RV) in a child after Mustard procedure.

A 7-year-old girl with a history of Mustard surgery performed at the age of 1, along with patch closure of a ventricular septal defect, presented with dyspnea on moderate exertion and fatigue. Upon admission, her brain natriuretic peptide (BNP) level was 65 pg/mL, and echocardiography revealed severe TR and reduced ejection fraction (EF) of the SRV (35%), a global longitudinal strain (GLS) of 9%, and a cardiac index (CI) of 1.9 L/min/m^2^. RV dimensions were increased, with an end-diastolic diameter (EDD) of 59 mm (Z-score of 4.91, Figure 1). Tricuspid annular plane systolic excursion (TAPSE) was found to be 9 mm. Magnetic resonance imaging (MRI) (Siemens Magnetom Avanto FIT, Germany) showed an EDD of 56 mm, an end-systolic diameter (ESD) of 40 mm, an indexed end-diastolic volume (EDV) of 127 mL/m^2^, an indexed end-systolic volume (ESV) of 64 mL/m^2^, and an EF of 36%. Given the patient’s clinical presentation and imaging findings, therapy for HFrEF was initiated. Medications included spironolactone 25 mg/day, torasemide 5 mg/day, carvedilol 6.25 mg/day, sacubitril/valsartan 50 mg/day, and dapagliflozin 5 mg/day. A summary of the preoperative period presented in Table 1. Over the course of one year, the patient showed significant improvement, with an increase in RV function (SVEF 45–48%), a CI of 2.5 L/min/m^2^, TAPSE of 16 mm, and a reduction in BNP to 25 pg/mL. Subsequently, the patient underwent mechanical valve replacement of the systemic atrioventricular (tricuspid) valve. Levosimendan infusion was added perioperatively (1 day before and after). The surgery lasted for 152 min of cardiopulmonary bypass, with 70 min of aortic cross-clamping (cold crystalloid cardioplegia). The postoperative course was uncomplicated, with a short period of mechanical ventilation (16 h) and a stay in the intensive care unit. On day three, the patient’s HF therapy was continued, and follow-up echocardiography before discharge (on the 10th day) revealed stable RV function, with an EDD of 45 mm, EF of 47–50%, and a CI of 2.9 L/min/m^2^. During the inpatient stay, the girl developed signs and symptoms consistent with acute cystitis, which was temporally associated with ongoing dapagliflozin therapy. The medication was subsequently discontinued, followed by targeted uroseptic treatment. Two years after surgery, the patient underwent follow-up evaluation at a pediatric rehabilitation center, which showed continued preservation of RV function (EF 47%) and good prosthetic valve function. Additionally, there was further improvement in the CI (3.2 L/min/m^2^) and GLS (15.4%). A summary of surgical procedures and the postoperative period presented in Table 2.

### 2.2. Case 2

Patient 2 was a 45-year-old female diagnosed with ccTGA. She had previously received a dual-chamber pacemaker due to spontaneous atrioventricular block. Over a period of 6 months, her symptoms, including dyspnea, congestion, and exercise intolerance, worsened. Upon admission, echocardiographic findings revealed EF of 27%, RV EDD of 6.2 cm, CI of 1.8 L/min/m^2^, TAPSE of 11 mm, severe mitral and tricuspid regurgitation, and mild pulmonary hypertension. QRS duration was about 140 msec. Laboratory results showed a BNP level of 1280 pg/mL and a creatinine level of 80 μmol/L. The patient’s treatment was initiated with sacubitril/valsartan (titrated to 200 mg/day), bisoprolol 2.5 mg/day, phosphocreatine, and diuretics (torasemide + spironolactone). A single dose of levosimendan (12.5 mg) was administered. After 1.5 weeks, the patient’s symptoms began to improve, and her EF increased to 32%. She was discharged on oral medications for 2 months. During this period, her functional status improved from III to II NYHA class, and by the end of the follow-up, her SRV EF had risen to 40%, with a CI of 2.8 L/min/m^2^, TAPSE of 16 mm, RV EDD of 5.8 cm, and BNP of 110 pg/mL, with stable, good kidney function. The patient received a second dose of levosimendan (12.5 mg) before surgery. Replacement of the systemic AV valve and subpulmonary AV valve was performed. The length of mechanical ventilation was 17 h. The surgery lasted for 220 min for cardiopulmonary bypass, with 145 min of aortic cross-clamping (cold crystalloid cardioplegia). Postoperative treatment included valsartan/sacubitril, phosphocreatine, spironolactone, and diuretics. The patient was discharged on day 11 post-operation. One year later, the patient reported good exercise tolerance, with an SRV EF of 43%, CI of 3.1 L/min/m^2^, RV EDD of 5.7 cm, and a BNP level of 80 pg/mL. Four years later, the patient’s BNP level was 147.3 pg/mL, and her SRV EF was 45%. She remained asymptomatic, with no evidence of HF or prosthetic valve dysfunction.

### 2.3. Case 3

Patient 3 was a 49-year-old man with ccTGA. At the time of admission, the patient demonstrated complete AV block and signs of SRV dysfunction, with a reduced SVEF of 37%, a CI of 2.2 L/min/m^2^, and markedly decreased contractility (derivative of pressure with respect to time (dP/dT) 437 mmHg/s). His BNP level was elevated at 543.4 pg/mL. QRS duration on ECG was 130 msec. Preoperative management included pharmacological therapy and pacing with temporary endocardial lead. The therapy included sacubitril/valsartan (50 mg/day), continuous dopamine infusion (5 μg/kg/min), torasemide (10 mg/day), and spironolactone (50 mg/day). After three weeks, SRV function was reassessed by echocardiography. During temporary pacing, control echocardiography showed that SVEF increased to 48%, dP/dT rose to 1100 mmHg/s, and CI improved to 3.1 L/min/m^2^. TAPSE was 18 mm, and tissue peak systolic velocity (s’) was 9.2 cm/s. Concurrently, BNP levels significantly decreased to 144.1 pg/mL. Due to the improvement in SRV function and the need for chronic pacing, surgical intervention was performed. The procedure consisted of mechanical prosthetic replacement of the systemic (tricuspid) valve and implantation of an epicardial dual-chamber pacing system. The surgery lasted for 186 min of cardiopulmonary bypass, with 100 min of aortic cross-clamping. The operation was uneventful, and the patient tolerated the procedure well. By postoperative day 14, at the time of discharge, the CI was 4.0 L/min/m^2^, and SRV EF was 40%. At discharge, the patient was maintained on sacubitril/valsartan (50 mg/day), bisoprolol (2.5 mg/day), and eplerenone (25 mg/day). This medical regimen was well tolerated, with no clinically significant adverse effects reported. At two-year follow-up, the patient demonstrated good exercise tolerance, with stable SRV EF and CI.

### 2.4. Case 4

**Figure 2:**
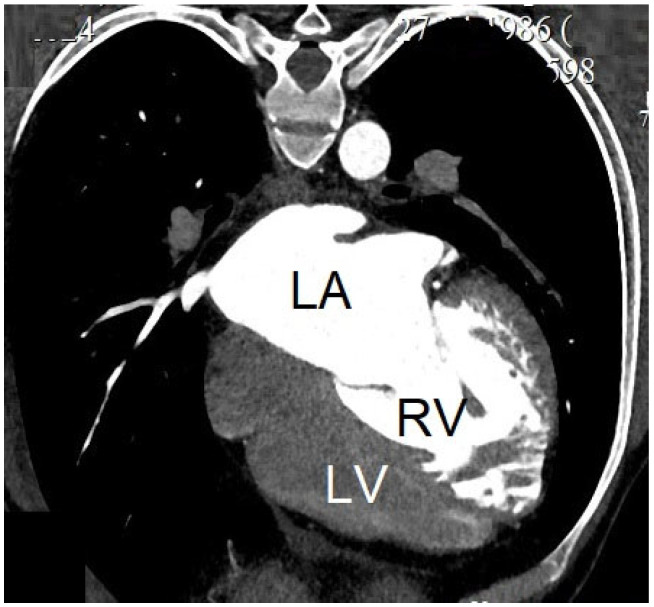
A computed tomography scan showed dilated systemic right ventricle (RV), dilated left atrium (LA), and “banana-shaped” left ventricle (LV) in an adult with ccTGA.

Patient 4 was a 37-year-old man with ccTGA complicated by ventricular tachycardia. On admission, echocardiography demonstrated a SVEF of 34%, indexed EDV of 280 mL/m^2^, CI of 1.49 L/min/m^2^, subpulmonary ventricular EF of 12% (Figure 2). The BNP level was 3732.3 pg/mL. Cardiac catheterization confirmed a low CI of 1.71 L/min/m^2^. Medical therapy was initiated with sacubitril/valsartan (25 mg/day), carvedilol (12.5 mg/day), eplerenone (25 mg/day), and torasemide (5 mg/day). After 17 months of optimized medical treatment, follow-up MRI revealed a significant improvement in SRV function, with an EF of 56% and a reduction in indexed EDV to 161 mL/m^2^. The subpulmonary ventricular EF increased to 65%. BNP level was 17.5 pg/mL. On echocardiography, the SVEF was 49%, TAPSE was 15 mm, dP/dT was 1213 mmHg/s, CI was 2.07 L/min/m^2^, and GLS was 16%. The patient subsequently underwent mechanical valve replacement of the systemic AV valve and repair of the subpulmonary AV valve. The surgery lasted for 191 min for cardiopulmonary bypass, with 124 min of aortic cross-clamping (cold crystalloid cardioplegia). He was transferred from the intensive care unit on postoperative day 2. Early postoperative echocardiography showed a transient decline in SVEF to 37%, which improved to 44% by discharge on postoperative day 25. The postoperative period was complicated by hemopericardium, requiring pericardial drainage on postoperative day 20 with clinical improvement. At discharge, echocardiography demonstrated an RV-indexed EDV of 120 mL/m^2^, EF of 44%, and CI of 3.3 L/min/m^2^. Six months after surgery, the patient remained in stable condition. Echocardiography revealed an SVEF of 49% and GLS improved to 18.1%, with no recurrent HF symptoms (NYHA class I–II).

## 3. Discussion

In this study, we showed a multi-stage approach in three adult patients and a child with SRV dysfunction and severe TR. Primary HF medication demonstrated an improvement in SVEF and CI. TV replacement was performed in all cases with good immediate and mid-term results. Further, SVEF remained >40% with continued therapy.

### 3.1. RV Dysfunction and TR Mechanisms

The systemic right ventricular dysfunction is a well-known complication in ACHD patients and is caused by many factors. The muscular architecture consists of one longitudinal (deeper) and one horizontal (superficial) layer. The unique anatomical characteristics of the SRV—including its heavily trabeculated morphology, predominant longitudinal fiber orientation, and complex subvalvular apparatus—may influence the progression of dysfunction and modify the response to medical therapy. These structural features contribute to altered mechanics under systemic pressure load and predispose the valve apparatus to dysfunction. Compensatory mechanisms lead to hypertrophy. In hypertrophic RVs, coronary artery supply may be insufficient, potentially contributing to the development of fibrosis and systolic dysfunction. Myocardial perfusion abnormalities are frequently observed following atrial switch procedures, particularly affecting the inferior wall. The perfusion defects occur more commonly in patients who underwent later surgical repair, and their extent strongly correlates with impaired biventricular function [2]. Diastolic dysfunction also significantly contributes to SRV impairment, although the precise mechanisms remain poorly understood. In TGA after atrial switch, it has been demonstrated that baffle rigidity can limit preload and stroke volume, particularly with tachycardia, leading to inadequate RV filling and symptoms of HF. Furthermore, conduction abnormalities and complete heart block requiring ventricular pacing are highly prevalent in ccTGA, affecting up to 50% of patients. Long-term left ventricular pacing can lead to pacing-induced dyssynchrony, thereby aggravating SRV dilation and progressive dysfunction [3,4].

The TR in the context of SRV arises from multiple additional mechanisms. Dilation of the tricuspid annulus resulting from right ventricular enlargement under chronic systemic pressure is considered a major contributor to TR in this population. Moreover, TV is often dysplastic (such as Ebstein-like displacement), and these structural abnormalities can cause TR as well [2]. Prieto et al. provided compelling evidence that intrinsic morphological abnormalities of the TV are stronger predictors of TR severity in ccTGA than SRV dilation itself, suggesting that anatomical malformations play a primary role, whereas SRV dilation may be secondary [5]. Additionally, a mismatch between the increased oxygen demand of the subvalvular apparatus, secondary to papillary muscle hypertrophy, and a multifactorial reduction in coronary flow reserve may further exacerbate valvular dysfunction. Finally, TR progression leads to RV dilation, dysfunction, and EF reduction. All these factors create a “chicken and egg dilemma”, meaning “which came first: TR or RV dysfunction?” Recent studies do not provide an exact answer, and in our experience, there were patients manifesting both with valvular insufficiency or with ventricular dysfunction.

### 3.2. TV Surgery in SRV Patients

In one of the largest studies (F. Mongeon, Mayo clinics, 2011), the authors analyzed results of TV replacement in 46 patients with ccTGA [6]. Patients were grouped according to their SVEF: >40% (*n* = 27), <40% (*n* = 19). Preoperative data showed that HF medications were commonly used, regardless of ventricular function. Only some patients received ACEi (63% in group 2) or ARB (5% in group 2), beta-blockers (16% in group 2), or spironolactone (4% in group 1 and 0% in group 2), while the most common drug was digoxin (79% in group 2). There is no data regarding the change in ventricular function due to medication and its role in postoperative complications. Early mortality was absent, but SVEF decreased during the follow-up (7–8 years), and the use of HF medications became more common than pre-operatively. Late mortality was 15% in group 1 and 37% in group 2. Preoperative variables that predicted late mortality or cardiac transplantation included SVEF, sub-pulmonary ventricular systolic pressure, NYHA functional classes III to IV, and atrial fibrillation. According to this data, we can conclude that approaches in HF management significantly changed over the past 15–20 years, and new studies assessing the effectiveness of new medication regimens in SRV patients are needed. In our study, patients had SVEF > 40% and I NYHA class during the follow-up period, indicating the positive effects of OMT [6].

Deng et al. reported results of the long-term outcomes of TV surgery in 57 patients with ccTGA. In all cases, SVEF was more than 40%, early mortality was 1.8%, and 10-year survival was 75.6%. During the follow-up after surgery, 7.0% patients had reduced SVEF (<40%). This study has no information about medication, but shows a significantly lower survival rate in patients with an RV EDD ≥ 60 mm (1 cm increment; hazard ratio, 3.22; 95% confidence interval, 1.23–8.4; *p* = 0.02). Also, 19.3% patients underwent TV repair with suboptimal results [7]. Limited opportunities for TV repair with a lower freedom of TR recurrence were reported in other studies as well [6,8,9]. Also, O. Gonzalez-Fernandez et al. concluded that concomitant TV replacement at the time of ventricular assist devices implant is associated with better early hemodynamic and echocardiographic results in patients with advanced failure of the SRV [10]. We performed TV replacement in all cases with good hemodynamic characteristics. Also, it can be assumed that preservation of the subvalvular structures at the time of surgery can improve long-term ventricular function and prognosis. But we did not find results of comparison between different surgical techniques due to TR replacement in such patients in the literature.

A recent case report described a successful transcatheter valve-in-valve implantation in a patient with systemic RV dysfunction and severely reduced EF (35%) and prior systemic AV replacement with a bioprosthetic valve. This and several other examples highlight the potential of less invasive approaches in high-risk patients, suggesting that transcatheter therapies may serve as a feasible alternative when conventional surgery poses significant risks [11,12].

Recent case reports describe successful transcatheter valve-in-valve implantation in high-risk patients with SRV dysfunction. Such approaches may offer advantages including lower perioperative risk, avoidance of repeat sternotomy, and applicability in patients with severely reduced EF. Although clinical experience remains limited, transcatheter strategies represent a promising alternative in selected cases.

### 3.3. Medication in SRV Patients

Medication strategies in patients with systemic RV are still unclear. The management of HF in patients with an SRV remains a complex and evolving challenge, as outlined in several key reviews published in recent years. Kutty et al. emphasized the lack of robust evidence guiding pharmacological therapy for this unique population, noting that most treatment strategies are extrapolated from left-ventricular HF models and often limited by systemic hypotension and conduction system disease [13]. Similarly, Carazo et al. highlighted the need for a multimodal, individualized approach, combining imaging, biomarkers, rhythm surveillance, and structural interventions to optimize outcomes. The use of standard neurohormonal blockade (iACE, beta-blockers, MRAs) is common but remains largely empirical due to a paucity of randomized controlled trials [14]. More recently, Lluri and Aboulhosn provided an updated perspective, stressing the high prevalence of arrhythmias and valvular dysfunction in SRV patients and discussing the growing interest in novel pharmacotherapies such as sacubitril/valsartan and iSGLT2, as well as device-based therapies including cardiac resynchronization therapy and implantable cardioverter-defibrillators [15]. Collectively, these reviews underscore the urgent need for targeted clinical trials and tailored management strategies in adults with SRV, whose long-term outcomes continue to be suboptimal despite advances in congenital heart disease care.

Ephrem et al. conducted a prospective observational study evaluating the safety and efficacy of ARNI in adult patients with an SRV. The study included 18 patients (mean age 40 years), all of whom had an SRV due to congenital heart disease, most commonly following atrial switch procedures for TGA. Over a median follow-up period of 13 months, sacubitril/valsartan was well tolerated, with no reported cases of symptomatic hypotension or deterioration in renal function. Importantly, a statistically significant improvement in functional status was observed, with the median NYHA class improving from III to II (*p* = 0.005). However, no significant changes were noted in objective parameters, including echocardiographic indices, exercise capacity, or laboratory biomarkers. The number of HF-related hospitalizations decreased (from nine pre-treatment to four post-treatment), though this trend did not reach statistical significance (*p* = 0.313). Despite the limited sample size and absence of a control group, the findings suggest that ARNI may offer a subjective clinical benefit in this high-risk population. The study supports further investigation of sacubitril/valsartan in SRV patients through larger, randomized trials [16].

Lebherz et al. (2022) analyzed long-term outcomes, therapeutic management, and SRV function in 380 adult patients [17]. The cohort included patients post-atrial switch and with ccTGA. Approximately one-quarter to one-third exhibited moderate to severe systolic dysfunction of the SRV, frequently accompanied by systemic AV regurgitation and arrhythmias. Pharmacological treatment predominantly involved iACE, angiotensin receptor blockers, beta-blockers, and MRAs, yet nearly 40% of patients received no HF therapy. Echocardiographic parameters and clinical symptoms were used to evaluate ventricular function and treatment response. Medication use correlated with more advanced HF symptoms and ventricular impairment, indicating treatment initiation in more symptomatic patients. These findings emphasize the strong need for standardized treatment protocols and prospective studies to improve management and outcomes in this vulnerable population [18].

Although robust evidence in SRV populations is still lacking, experimental studies suggest that neurohormonal modulation may promote favorable molecular and structural changes in the pressure-loaded RV. Observational data in SRV patients indicate potential improvements in functional class and stabilization of ventricular function under modern HF therapy, supporting the hypothesis of early reverse remodeling, which may contribute to the preoperative improvement observed in our cohort [19].

## 4. Conclusions

The case series showed the efficiency of heart failure medication in patients with SRV dysfunction and severe tricuspid regurgitation. Preoperative optimization leads to improved ventricular function and may enhance surgical outcomes. The short ICU stay and preserved ventricular function during the follow-up period confirm the considered strategy. The small cohort size and absence of a control group are the main limitations of the study.

## Figures and Tables

**Table 1:** A summary of the preoperative period.

N	Age/ Sex	Initial CHD	Previous Procedures	Admission State	Medication	Treatment Period	Preoperative State
1	8/f	TGA	Mustard procedure	BNP 55 pg/mL, SVEF 35%, CI 1.9 L/min/m^2^, severe TR, GLS 9%, TAPSE 9 mm	levosimendan, ARNI, iSGLT2, carvedilol, MRAs, diuretics	1 yr 4 mos	BNP 25 pg/mL, SVEF 45–48%, CI 2.5 L/min/m^2^, TAPSE 16 mm
2	45/f	ccTGA	Pacemaker implantation	BNP 1218 pg/mL, SVEF 27%, CI 1.8 L/min/m^2^, severe TR, severe MR, TAPSE 11 mm	levosimendan, ARNI, bisoprolol, MRAs, diuretics, phosphocreatine	2 mos	BNP 110 pg/mL, SVEF 40%, CI 2.8 L/min/m^2^, TAPSE 16 mm
3	49/m	ccTGA, 3°AV block	-	BNP 543.4 pg/mL, SVEF 37%, severe TR, CI 2.2 L/min/m^2^, dP/dT 437 mmHg/s	dobutamine, ARNI, MRAs, diuretics	3 wk	BNP 144.1 pg/mL, SVEF 48%, CI 3.1 L/min/m^2^, dP/dT 1100 mmHg/s, TAPSE 18 mm,
4	37/m	ccTGA, VT	-	BNP 3732 pg/mL, SVEF 34%, CI 1.49 L/min/m^2^, severe TR	ARNI, carvedilol, MRAs, diuretics	1 yr 5 mos	BNP 17 pg/mL, SVEF 49%, CI 2.07 L/min/m^2^, TAPSE 15 mm, E/e’ 7, dP/dT 1213 mmHg/s, GLS 16%.

3°AV block—complete atrioventricular block; ARNI—angiotensin receptor neprilysin inhibitors; BNP—brain natriuretic peptide; CI—cardiac index; ccTGA—congenitally corrected transposition of the great arteries; iSGLT2—inhibitors sodium-glucose cotransporter 2; f—female; m—male; MR—mitral regurgitation; MRAs—mineralocorticoid receptor antagonists; SVEF—systemic ventricle ejection fraction; TAPSE—tricuspid annular plane systolic excursion; TGA—congenitally corrected transposition of the great arteries; TR—tricuspid regurgitation; VT—ventricular tachycardia.

**Table 2:** A summary of surgical procedures and the postoperative period.

N	Surgery	ICU Period	Complications	Postoperative Period	Discharge Data	Follow-Up
1	mechanical SAVV replacement	1 day	cystitis	10 days	SVEF 47–50%, CI 2.9 L/min/m^2^	1 year—SVEF 47%, CI 3.2 L/min/m^2^, GLS 15.4%
2	mechanical SAVV replacement and BP-AVV replacement	2 days	hydrothorax	11 days	SVEF 40%, CI 3.0 L/min/m^2^	18 mos—BNP 80 pg/mL, SVEF 43%, CI 3.1 L/min/m^2^ 4 years—BNP 147 pg/mL, SVEF 45%
3	mechanical SAVV replacement and implantation of an EPS	3 days	-	14 days	SVEF 40%, CI 4.0 L/min/m^2^	-
4	mechanical SAVV replacement and repair of the subpulmonary atrioventricular valve	2 days	hemopericardium	25 days	SVEF 44%, CI 3.3 L/min/m^2^	6 mos— SVEF 49%, GLS—18.1%

BP-AVV—bioprosthetic subpulmonary atrioventricular valve; CI—cardiac index; ICU—intensive care unit; EPS—epicardial pacing system; GLS—global longitudinal strain; HF—heart failure; SAVV—systemic atrioventricular valve; SVEF—systemic ventricle ejection fraction.

## Data Availability

The datasets used and/or analyzed during the current study are available from the corresponding author on reasonable request.
